# Control of chronic *Strongyloides stercoralis* infection in an endemic community may be possible by pharmacological means alone: Results of a three-year cohort study

**DOI:** 10.1371/journal.pntd.0005825

**Published:** 2017-07-31

**Authors:** Russell Hays, Adrian Esterman, Robyn McDermott

**Affiliations:** 1 Kutjungka Clinics, Kimberley Aboriginal Medical Services Council, Broome, Australia; 2 Centre for Research Excellence in Chronic Disease Prevention, The Cairns Institute, James Cook University Cairns, Smithfield, Australia; 3 Sansom Institute of Health Service Research and School of Nursing and Midwifery, University of South Australia City East Campus, Adelaide, Australia; 4 Public Health Medicine, Centre for Chronic Disease Prevention, Australian Institute of Tropical Health and Medicine, College of Public Health, Medical and Veterinary Sciences, James Cook University Cairns, Smithfield, Australia; QIMR Berghofer Medical Research Institute, AUSTRALIA

## Abstract

**Objectives:**

To assess the effect of treatment with ivermectin on the prevalence of *S*. *stercoralis* infection in an Australian Aboriginal population over a three year period, and to assess the validity of using a lower ELISA cut-off in diagnosis.

**Methods:**

A three-year cohort study of 259 adult Australian Aboriginals living in a remote community in northern Australia. *S stercoralis* infection was diagnosed using commercial ELISA testing, and employed a lower threshold for treatment than that recommended. Follow up was conducted at 6 months and 3 years following ivermectin treatment.

**Findings:**

Treatment with ivermectin was highly effective and resulted in a sustained fall in the prevalence of infection in the study group (Initial prevalence 35.3%, 3 year prevalence 5.8%, McNemar’s chi^2^ = 56.5, p<0.001). Results of treatment suggested use of a lower ELISA threshold for treatment was valid in this setting. Follow up identified a small group of subjects with persistently positive ELISA serology despite repeated treatment.

**Interpretation:**

Control of *S*. *stercoralis* infection in this cohort appears to be feasible using pharmacological treatment alone.

## Author summary

Infection with the worm *Strongyloides stercoralis* is common throughout the developing world, and in some resource poor communities living within developed societies, such as the Aboriginal communities of northern Australia. It is generally agreed that reliable diagnosis of this infection is possible by blood tests, and that the medication ivermectin represents the best available treatment, however questions remain over how to best control and eliminate the infection in areas where it is common. Strongyloides infections may be asymptomatic, and may persist indefinitely without the need for re-infection. The worm is transmitted by contact with contaminated soil. Suggested strategies for control have therefore included mass administration of ivermectin in affected areas, and environmental measures to prevent the contamination of soil. In this study we follow up a group of subjects living in an endemic community three years after they were tested and treated for strongyloides infection. We find a persisting low prevalence of infection in this group in the absence of any environmental changes or further treatment, suggesting that control of the infection in this community might be achieved through simple case finding and treatment alone. In addition, we suggest the use of a lower cut-off value for serological testing in these communities, in order to avoid missing cases of infection.

## Introduction

Infection with the soil-transmitted helminth (STH) *Strongyloides stercoralis* is common in both the developing world, and in underprivileged communities in developed countries. Prevalence is frequently estimated at 30–100 million cases world wide, but due to difficulties with diagnosis, the frequent absence of symptoms in chronic cases, and the lack of extensive screening for the infection, this may be an under-estimate [1]. The major medical impact of infection is thought to be the occurrence of strongyloides hyperinfection syndrome, which occurs predominantly in infected adults who become immunosuppressed, whether by iatrogenic means or otherwise [2].

It is increasingly acknowledged that *S*. *stercoralis* infection represents a significant public health challenge and that action is required to deal with the problem [3], however there is no general agreement as to what these measures should be. Some advocate the need for extensive environmental management of the problem to interrupt transmission in endemic communities [4, 5]. They point out that pharmacological treatment can have side effects, cannot prevent re-infection from environmental sources of the infection, and that resistance to ivermectin may develop with excessive use. Others emphasize the efficacy and safety of treatment with ivermectin, and advocate pharmacological control, with possible mass drug administrations (MDA) in communities that have a high prevalence of infection in accordance with programs currently employed to control other soil transmitted helminth and vector borne nematode infections [1]. They point out that free-living forms of the parasite are relatively transient, human beings constitute the major reservoir of infection, and while some resistance has been noted in veterinary settings, to date there is no evidence of resistance in human infection. Still others have advocated a combination of these approaches, employing case finding and treatment with ivermectin, in association with public health measures such as improved sanitation [6].

A measure of success has been reported to date, with studies employing both approaches reporting positive results in control of the infection. [6, 7]

It should be pointed out that the optimal measures for control of strongyloides infection may differ from situation to situation, depending upon local factors such as rainfall and vegetation, the importance of agricultural practices in transmission, and the presence or otherwise of adequate health infrastructure. Because of the chronic nature of this infection and its capacity for sustained auto-infection within individuals, it is possible to have a high prevalence of infection both in communities where conditions allow for high transmission rates of the infection, and equally in communities where transmission is lower, but there are a high number of chronic, untreated infections.

The Aboriginal communities of northern Australia are home to some of the highest measured prevalence of this infection, with rates of 30–40% reported [8].

Oral ivermectin is now accepted as the treatment of choice for this condition, but disagreement persists over the need for wide spread testing and elimination of the infection. Attention has been drawn to some of the barriers preventing control of infection in these communities, including difficulties with diagnosis and lack of adequate follow up of treated cases [9].

Direct parasitological diagnosis of infection and other coprological methods are generally not feasible in these isolated communities, and are known to have a low sensitivity even when performed under optimal conditions. ELISA testing is now generally accepted as being sensitive and specific enough for use in both clinical and research settings [1, 10]. Concerns over cross reactivity with other helminth infections have less relevance in the Australian setting where these infections are not found. In practical terms in the Australian setting this equates to testing using one of several commercially available ELISA tests. The reference ranges for these tests were developed in relatively low prevalence populations and were intended to prevent false positive results in situations where the infection is uncommon. For example, the unreferenced product information for one such test (DRG laboratories) advises clinicians that normal ranges may need to be interpreted “in the context of local settings”. Anecdotally, it is reported that for this reason, clinicians working in communities where the infection is known to be endemic frequently treat patients whose ELISA results fall under the published range, in an attempt to avoid missing infections. To date there have been no attempts to validate this approach, and studies of treatment efficacy have mostly been characterized by limited and poor follow up of treated cases [8].

The current study comprises a three year follow up of a cohort of patients tested and treated for strongyloides infection in an Aboriginal community in northern Australia as part of a study into the relationship between *S*. *stercoralis* infection and type 2 diabetes mellitus [11]. It is the first study to examine the long term outcome for adults tested and treated for the infection, who continue to live in an endemic community in the absence of any attempt at environmental manipulation.

Furthermore, as the original cohort was established using a revised (and lower) cut off for ELISA positivity, there can now be an attempt to validate the use of a lower ELISA threshold for treatment by looking at response to treatment and long term outcomes in these patients.

## Methods

### Study population

The study was conducted in three aboriginal communities located within a 100 km radius of each other in the far north Kimberley region of Western Australia. The communities are isolated, with the nearest hospital and laboratory services being 800km away. Prior to the commencement of this study, testing and treatment for *S*. *stercoralis* infection had not taken place in any systematic manner.

The cohort comprised 259 adult aboriginals attending the medical facilities in these communities, and was originally established from April 2012 to December 2013 as part of a study into the relationship between *S*. *stercoralis* infection and type 2 diabetes mellitus (T2DM). The protocol for this study has been published previously [11].

Patients were offered testing and treatment for *S*. *stercoralis* infection on an opportunistic basis when attending the clinics. No attempt was made to screen patients on the basis of presenting symptoms, as it was felt that there was little evidence for the presence of reliable symptoms in chronic strongyloidiasis in an endemic setting. Recent results from a study conducted in an Aboriginal community would seem to support this [12]. Patients who were normally resident outside the communities were excluded, as were patients who had received prior treatment with ivermectin, without serological testing for *S*. *stercoralis*.

### Serological testing for *S stercoralis*

Diagnosis of *S*. *stercoralis* infection was established purely through serological means as direct parasitological studies would have been logistically difficult in this setting, and would likely have lacked sensitivity [13].

ELISA testing was carried out at a reference laboratory utilizing a commercial *S*. *stercoralis* ELISA kit (DRG laboratories) that tests for the presence of IgG antibodies. All positive results were subsequently tested in parallel and the revised value used in analysis.

The normal ranges (in units of absorbance) provided by the laboratory for this test were

: < 0.20 –Negative

: 0.20–0.40 –Equivocal

: > 0.40 –Positive

A prior survey in the study community had suggested that a prevalence of infection greater than 30% was likely [14] and therefore, in order to reduce the possibility of false negative results, a modified normal range was employed for diagnosis. All results greater than 0.30 were considered as “positive”, and were treated. All results less than 0.30, including those less than 0.2, were considered “equivocal” from the point of view of follow up, and were placed on recall for repeat testing in 6 months.

### Treatment and follow up

All patients returning positive results according to the protocol were treated with 2 doses of ivermectin 0.2mg/kg, given 2 weeks apart under direct observation.

All 259 patients in the study were then recalled after 6 months for repeat serological testing. The results of 6 month testing have been published previously, as part of a study into the relationship of treatment outcomes and T2DM [15].

Any subjects found to be ELISA positive at 6 months were recalled for repeat treatment with a single dose of ivermectin 0.2mg/kg. An audit of patient records shows that of the 29 subjects who remained ELISA positive at 6 months, 24 received an extra dose of ivermectin at this time, while 5 did not.

Routine follow up of patients was then conducted by the individual clinics. Clinic records show that a further 5 subjects received a fourth dose of ivermectin, for presumed strongyloides infection, during the subsequent 2½ years.

All subjects were finally recalled approximately 3 years after initial testing for repeat ELISA testing and metabolic assessment. Any subjects found to be ELISA positive at this time were treated with a further dose of ivermectin 0.2mg/kg. Three year follow up also involved an assessment of the subject’s metabolic status as part of an ongoing study into the relationship between strongyloides and T2DM, the results of which will be published separately [16].

### Treatment outcomes

Treatment outcome in this study was evaluated in two ways. Firstly, “treatment success” was defined as a fall in ELISA serology to < 0.30 at follow up. “Treatment effect” was defined as a ratio of post treatment ELISA to pre-treatment ELISA of less than 0.60.

As a modified normal range was used in this study to identify positive cases, at follow up it was possible to compare two groups; those whose initial serology was above the established positive range of ≥ 0.40 (designated “high titre”), and those whose initial serology was in the range between ≥ 0.30 and< 0.40 (designated “low titre”) These groups were compared in terms of treatment success and treatment effect on the basis that if those in the low titre group represented true infections with *S*. *stercoralis* (rather than false positives) then treatment outcomes should be similar to those achieved in the high titre group. Additionally outcomes for the two groups were compared in terms of the average ELISA serology of both groups at 3 years follow up, on the basis that if both groups represented true infections, and both groups were treated in the same way, then the two groups should be similar in terms of outcomes at 3 years.

### Assessment of “non-responders”

During the course of follow-up it became apparent that a small number of subjects continued to return positive ELISA results after three years of follow up, despite repeated treatment with ivermectin (designated “non-responders”). Clearly it is of interest as to whether these non-responders represent true treatment failure or re-infection, or whether they are simply continuing to produce a positive ELISA test in the absence of ongoing infection. To aid in the assessment of these subjects, additional testing was carried out, including serological testing for HTLV-1 virus, serum IgE, FBE, and a multivalent faecal PCR test for *S*. *stercoralis* targeting a 101bp region of the 18S gene (Western Diagnostic Pathology, Myaree) [17]. Molecular testing of faeces specimens was employed, as it is logistically easier to achieve from an isolated location. Studies suggest that, although still less sensitive than serology, it has improved sensitivity over direct parasitological methods[18].

### Statistical analysis

McNemar’s chi-square test for correlated proportions was used to compare prevalence rates at the different time points. Comparison of mean age and BMI and change in eosinophil count between responders and non-responders was undertaken using independent samples t-tests. No attempt was made to impute data for those lost to follow up. Logistic regression was used to determine differences in outcome for high titre and low titre cases.

### Ethics statement

The protocol for this study was approved by the Kimberley Aboriginal Health Planning Forum. Ethical approval for the study was obtained through the Western Australian Aboriginal Health Ethics Committee (WAAHEC) in 2014 (HREC Reference 515). All subjects were over 18 years of age and provided informed verbal consent. Use of verbal consent was approved by WAAHEC as no additional tests or treatments were required other than those dictated by the current best management guidelines for this condition, and due to variable rates of literacy in the study community. Consent was recorded electronically in the subject’s permanent medical record.

## Results

### Treatment outcomes

Demographic data for the 259 patients originally enrolled in the study are given in Table 1.

**Table 1:** Demographic data.

		Not treated		Treated		Overall		Sig.
		N	%	N	%	N	%	P
**Age**	<30	18	10.7	17	18.7	35	13.5	0.141
	30–39	64	38.1	26	28.6	90	34.8	
	40–49	44	26.2	20	22.0	64	24.7	
	50+	42	25.0	28	30.8	70	27.0	
	Total	168	100.0	91	100.0	259	100.0	
**Sex**	Male	69	41.1	37	40.7	106	40.9	0.949
	Female	99	58.9	54	59.3	153	59.1	
	Total	168	100.0	91	100.0	259	100.0	

**Fig 1:**
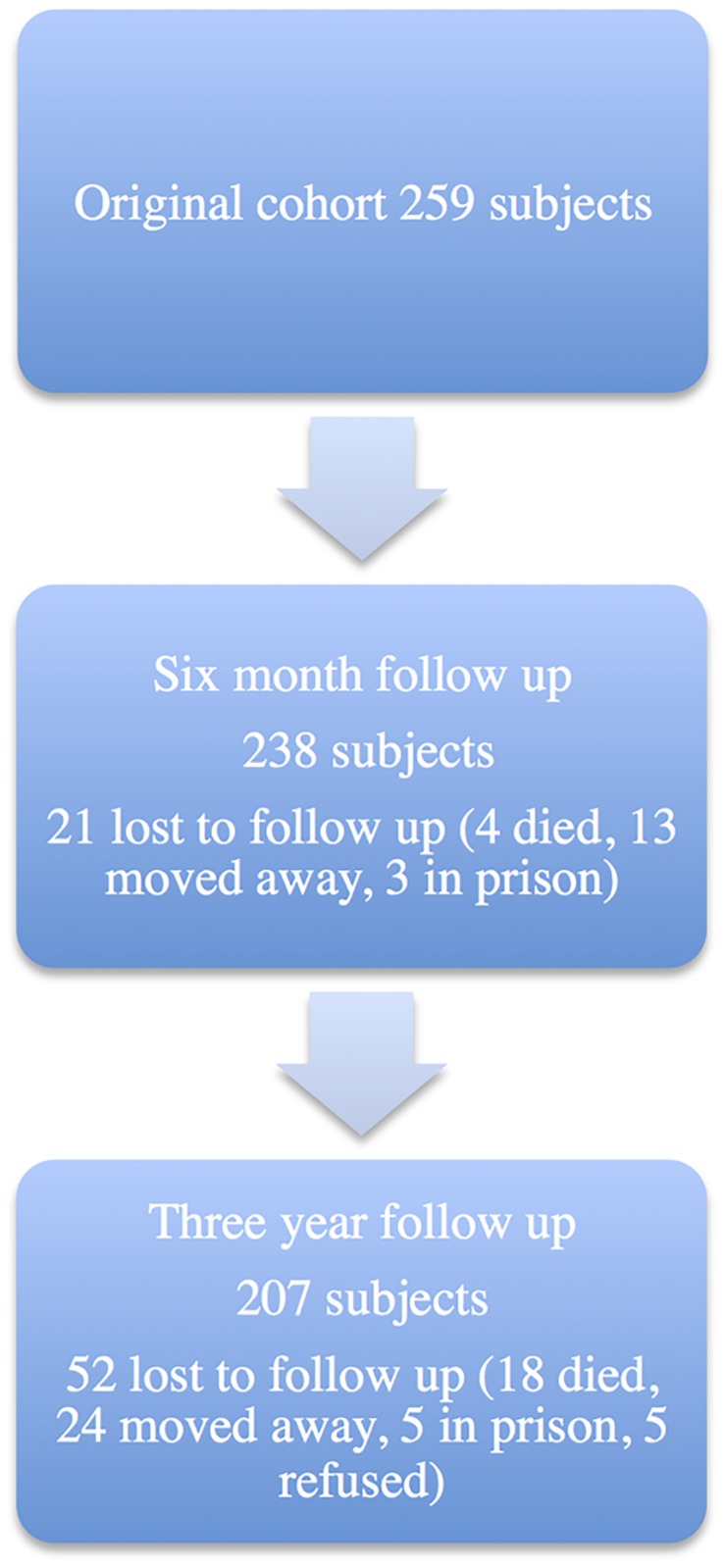
Outcome of follow up at three years.

Successful follow up was achieved in 207 of the original cohort at 3 years, with 52 subjects lost to follow up as outlined in Fig 1. Those lost to follow up were similar in terms of infection status (36.5% vs. 35.2%, p = 0.85), with a tendency to be female (62% vs. 48%; p = 0.071). All other baseline comparisons were similar.

**Fig 2:**
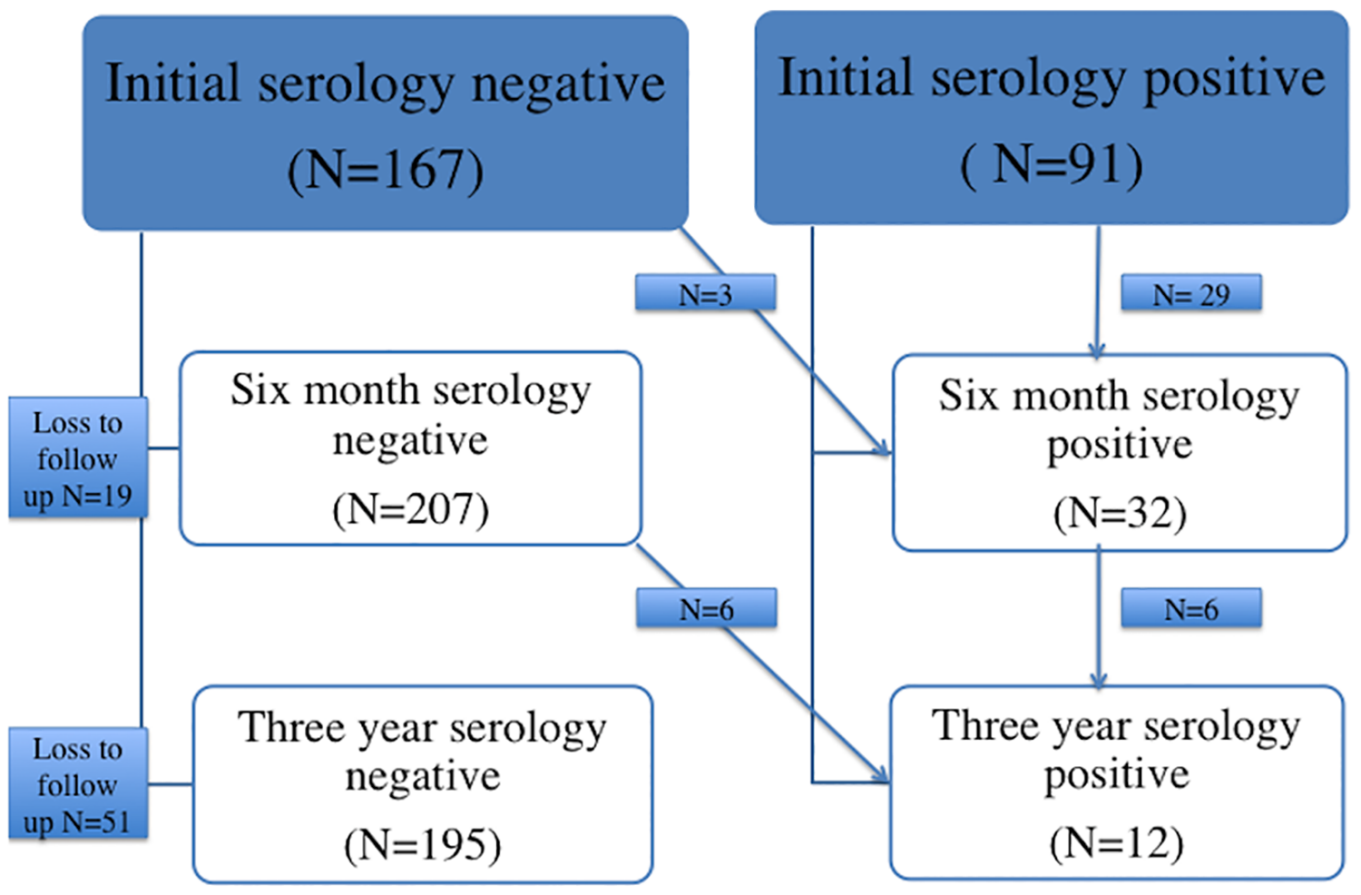
Treatment outcome at three years. *One patient who tested positive to *S*.*stercoralis* but left the community prior to treatment, has been excluded from analysis.

Fig 2 gives a graphical illustration of the outcome in terms of serology for those followed up at 6 months and 3 years. Treatment with ivermectin was highly effective, with the prevalence of ELISA positive subjects falling from an initial 35.3% to 13.4% (McNemar’s chi^2^ = 46.6, p<0.001) at 6 months and 5.8% (McNemar’s chi^2^ = 56.5, p<0.001) at 3 years, respectively.

Only three new cases were diagnosed in the first 6 months of follow up, with a further three in the period up to 3 years. Three cases that had returned to negative at 6 months were found to be ELISA positive again at 3 years. It should be noted that it is not possible to ascertain whether these three cases represent relapse of existing infection, presumably through the process of “auto-infection “which is known to occur in chronic strongyloidiasis, or re-infection from environmental sources. The details of these subjects are summarized in Table 2.

**Table 2:** New cases at follow up.

Subject	Age	Initial ELISA	Six month ELISA	Three year ELISA
**Female**	53	0.19	0.30^	0.12*
**Female**	43	0.15	0.43^	0.20*
**Female**	30	0.18	0.30^	0.22*
**Male**	43	0.22	-	0.34^
**Male**	69	0.15	0.24	0.33^
**Female**	29	0.14	0.03	0.40^
**Female**	73	0.48^	0.28	0.37^
**Female**	70	0.51^	0.29	0.37^
**Male**	43	0.35^	0.27	0.32^

*After treatment at 6 months follow up.

^-^ Subject was lost to follow up at 6 months.

^Positive results.

All new cases at 6 months and 3 years involved only a marginal increase in ELISA, with only two cases rising above the accepted positive range of 0.40, and the others deemed positive due to the use of a reduced cut-off in this study. Additionally, the three cases that “relapsed” after cure at 6 months in fact only dipped marginally below the cut-off of 0.30, before returning to marginally above.

An analysis of treatment outcomes at 6 months with respect to treatment failure has been published previously [15].

### Non-responders

Of the 12 positive cases at 3 year follow up, 9 represent “treatment failure”, with these subjects remaining ELISA positive throughout follow up, despite repeated treatment with ivermectin. The details of these subjects are presented in Table 3 along with the results of the further tests conducted. Comparisons of mean age, BMI and eosinophil counts with the subjects who responded to treatment are included.

**Table 3:** Details of non-responders.

	Age	Initial BMI	Initial ELISA (abs.)	Six month ELISA	Final ELISA	Initial eosin. (x10^9^)	Final eosin.(x10^9^)	HTLV-1	IgE (kU/ml)	Fecal PCR
**1 Male**	75	30.37	0.65	0.45	0.45	0.59	0.13	Neg	- *	-
**2 Female**	58	32.94	0.67	0.59	0.4	0.37	0.23	Neg	323	-
**3 Female**	73	29.6	0.48	0.28	0.37	0.57	0.39	Neg	87	neg
**4 Female**	70	46.05	0.51	0.29	0.37	0.47	1.06	Neg	-	neg
**5 Female**	45	29.11	0.59	0.34	0.32	0.36	0.17	Pos	1010	-
**6 Male**	31	38.85	0.38	0.31	0.32	0.51	0.33	Neg	223	neg
**7 Female**	40	56.01	0.73	0.58	0.46	0.39	0.42	Neg	137	neg
**8 Female**	46	34.08	0.35	0.34	0.34	0.94	0.62	Neg	1187	neg
**9 Male**	43	24.4	0.35	0.27	0.32	0.85	0.62	Neg	214	neg
**“Non-responders” average**	53.4	35.7					Av. Change In eosin. 0.12			
**“Responders” average**	41.8	28.1					Av. change in eosin. 0.41			
**Comparison**	Diff 11.7 p = 0.024	Diff 7.6 p = 0.005					Diff -0.29 p = 0.17			

*Missing values are indicated by–

Analysis shows them to be significantly older (difference 11.7 years, 95% C.I. 1.6–21.8, p = 0.024), and heavier (BMI difference 7.6, 95% C.I. 2.4–12.9, p = 0.005) than those who responded to treatment, with a smaller fall in eosinophil count after treatment, which did not reach statistical significance (difference -0.29, 95% CI -0.71–0.13, p = 0.167). Four of the seven subjects tested had IgE levels in the normal range.

### Comparison of outcomes for low titre and high titre cases

Table 4 shows a comparison of the outcomes for the low titre (initial serology ≥ 0.30 and< 0.40) and high titre (initial serology ≥ 0.40) groups.

**Table 4:** Outcome for “low titre” subjects.

	“Treatment success” (% success)	“Treatment response” (% effective)	Post treatment average ELISA (95% C.I.)
**“Low titre” ELISA (N = 16)**	13 (81.2)	10 (62.5)	0.146 (0.08–0.21)
**“High titre” ELISA (N = 58)**	52 (89.7)	54 (93.1)	0.143 (0.11–0.17)
**Analysis**	O.R. 2.0 (95% C.I 0.44–9.08, p = 0.369	O.R. 8.1 (C.I. 1.9–34.0, p = 0.004) Adjusted O.R.* 2.98(C.I. 0.46–19.31, p = 0.25)	Difference = 0.003 (C.I. -0.07–0.06, p = 0.92)

* Adjusted for initial ELISA titre

As can be seen, the difference between the two groups in terms of treatment success is not statistically significant, and they are very similar in terms of average ELISA titre at 3 year follow up. There is a significant difference when compared in terms of “treatment effect”, but this not significant when adjusted for initial ELISA titre.

## Discussion

The results of this study suggest that control [19] of *Strongyloides stercoralis* infection in this adult Aboriginal population could be achieved by case finding and treatment alone. A marked and sustained reduction in infection rates was seen in the study group without the need for environmental manipulation, or repeated follow up and treatment beyond the current best practice recommendations. The advantage with this strategy is that it requires no change in the current diagnostic or treatment guidelines, simply a sustained clinical response from those agencies providing health care in these communities.

The conditions present in Aboriginal communities differ in several ways from those in populations elsewhere in the world, where different strategies for control of *S*. *stercoralis* have been employed, and therefore the findings in this study may not be easily generalized.

Rates of *S*. *stercoralis* infection, as with other soil-transmitted helminths, are thought to rise throughout childhood, peaking in late adolescence or early adulthood, and remaining relatively constant through adult life [20]. In the study community it is likely that most infections occur in childhood and adolescence as a result of recreational activities around watercourses.

A study conducted in Cambodia employed a strategy that involved both case finding and treatment and attempts to improve sanitation, and reported success in control of infection over a two year follow up [6]. The incident rate of new infection was higher in this setting when compared to our study, and transmission was facilitated by agricultural practices that are not a factor in Aboriginal communities. Furthermore, the standard of housing and hygiene facilities in the Cambodian communities was lower and more variable.

It is not the case that improvements in hygiene were not necessary for control of STH infections in Aboriginal communities, rather that most of the necessary changes may have already occurred over the past few decades. Studies of the prevalence of hookworm infection in northern Australia suggest that control has been achieved through both pharmacological means and improvements in housing [21, 22]. Current living conditions in Aboriginal communities remain poor by broader standards, but in the current study community all individuals are, at a minimum, housed in purpose built dwellings with toilet facilities.

Mass administration of ivermectin has been advocated for control of *S*. *stercoralis* in high prevalence communities. This strategy appears to have been effective in Central American studies examining the administration of ivermectin for control of onchocerciasis, with sustained falls in the prevalence of *S*. *stercoralis* infection in areas subject to regular MDA compared to those who are not [7].

Such MDA programs would potentially have secondary health benefits in Aboriginal communities in terms of a reduction in *Sarcoptes scabiei* infections, with a subsequent reduction in Group A streptococcal infection and its sequelae of rheumatic fever and post-streptococcal glomerulonephritis. Trials of this approach have been conducted in Northern Australia with variable results [3]. The results of two MDA’s of ivermectin for the treatment of strongyloides in an Australian Aboriginal community have recently been published in this journal. A marked reduction in the prevalence of strongyloides at 6 and 18 months was demonstrated using this approach [12]. Such a strategy however, would require the active participation of affected communities and public health authorities alike, and agreement on the need for such a program has not yet been reached [23].

### Clinical effects of treatment

The principal aim of this study was to determine the effectiveness of a “case finding and treatment” approach to controlling *S*. *stercoralis* infection in an Aboriginal community. No attempt was made in our study to characterize the initial symptoms of subjects with chronic strongyloidiasis, and therefore there are no data with regard to improvements in the well-being or health of the community in response to treatment. A recent, much larger trial published in this journal did examine the benefits in terms of symptom control of MDA for strongyloides, and found no evidence of a significant change in symptom profile [12]. Most discussion of the benefits of controlling strongyloides infection has centred on the prevention of hyperinfection in immunocompromised individuals, and such patients are routinely treated on a purely empirical basis in parts of northern Australia [12]. No cases of hyperinfection were recorded in the study community during the course of the study.

Our prior study identified T2DM as a risk factor for failure for treatment of *S*. *stercoralis* [15]. No meaningful association of T2DM with treatment outcome could be identified at 3 years because of the very low number (9 subjects, 3 of whom had T2DM) failing to seroconvert. Extensive data however have been recorded in regard to the metabolic outcome for the subjects in this study and these will be reported on separately [16]. In summary, the data showed a differential effect of treatment for *S*. *stercoralis* on the metabolic outcomes for patients depending on their diabetic status. The incidence of worsening glucose metabolism in non-diabetic patients (new cases of T2DM or glucose intolerance) was higher in the treatment group (RR 3.75, CI 1.06–13.2, p = 0.04). At the same time improving diabetic control as measured by HbA1c was noted in the treated diabetic group (Diff = -1.03, p = 0.009). While the low numbers in this trial mean that this result should be interpreted cautiously, they are in agreement with another recently published study into the effect of albendazole treatment for STH infection on subsequent insulin resistance [24]. The possible adverse effect of treatment on glucose metabolism is of importance when considering the case for MDA in strongyloides. As a public health measure, MDA does not allow for consideration of the individual clinical state of the subjects being treated, and therefore intervention to monitor and improve the glucose metabolism of individuals at risk of developing T2DM is not possible.

Limitations of the present study include its reliance on serological means alone for diagnosing *S*. *stercoralis* infection, as direct parasitological tests are required to ensure maximum specificity. However, as already noted, direct methods lack sensitivity, are difficult to employ in remote locations, and can over estimate the response to treatment [10].

Several caveats need to be raised on the effectiveness and feasibility of a case finding and treatment strategy. Firstly, the current study deals only with an adult population, and little or nothing is known of the situation with respect to the prevalence of infection, and effectiveness of treatment in the paediatric population. Logically it would seem likely that infection and re-infection rates might be higher in children, where hygiene practices may be less rigorous and activities where infection could occur more common. Disagreement on the need for elimination in adults still exists, and this may be even more so in the case of children. Few clear severe adverse health effects of chronic infection have been demonstrated in children, and the major health consequence in adults- the occurrence of strongyloides hyperinfection syndrome in immunosuppressed patients-has been reported only rarely in children [2, 25]. It may therefore be reasonable to employ a regimen where cohorts of children are screened and treated as part of health screening procedures as they reach adulthood. Furthermore, as noted above, there is increasing evidence that pre-existing helminth infections may have beneficial effects on metabolic parameters in later life, and this potential effect may need to be taken into consideration when considering treatment in populations where type 2 diabetes mellitus is extremely prevalent [26].

Case finding and treatment also relies on the accurate and sensitive diagnosis of infection by the means currently available, as large numbers of false negative tests would be a problem for effective control. The similarity in response to treatment between the “low” and “high “titre groups in this study suggests that it may be appropriate to treat individuals whose ELISA values fall just below the positive range when working in a high prevalence community. Further research into the appropriate ELISA normal range for these populations may be of benefit.

Accurate follow up of treated cases would also be important in a case finding and treatment strategy. A significant number (9 cases, 9.8%) in this study remained ELISA positive throughout the study period despite adequate treatment, and it is important to know whether these represent true infections. Repeated re-infection of these subjects would seem unlikely as an explanation given the low overall incident rate of infection in the population. Resistance of the worm to treatment is certainly a possibility, although this is yet to be reported in a human population [4]. Alternately, it is possible that a persistent antibody response has developed in these individuals in the absence of ongoing infection, in a situation analogous to the “serofast” response sometimes seen in treponemal infection [27]. Further investigation of the 9 subjects in this study has not served to settle the matter. Faecal PCR studies were conducted in 6 cases and were negative, suggesting an absence of infection, or at least an absence of larval shedding at the time of testing. Eosinophilia is known to be an unreliable indicator of infection [28], however it is perhaps of note that eosinophilia rates that differed prior to treatment in the responding and non-responding subjects were almost identical following treatment. HTLV-1 infection is known to pre-dispose to severe and persistent strongyloides infection [29], but was present here in only one individual. IgE levels are commonly elevated in chronic infection but are reported to be normal in some cases, particularly in those co-infected with HTLV-1 and in the elderly [30]. Only two of the patients in this group had elevated levels of IgE at follow up. On balance, the further tests conducted on the 9 non-responder cases in this study do not support the presence of on-going infection with *S*. *stercoralis*. Clearly further studies to characterize the immune response in subjects such as these would be of interest, as it is known that changes in relative levels of inflammatory and anti-inflammatory cytokines occur in patients following successful treatment for *S*. *stercoralis* infection [31].

It should be noted that if the 9 non-responder subjects are excluded from analysis on the presumption that they are no longer infected, then the prevalence of *S*. *stercoralis* in this cohort 3 years after treatment falls to just 1.4%, underlining once again the success of this treatment strategy.

### Conclusion

In conclusion, this study provides evidence that control of chronic *S*. *stercoralis* infection in an adult Aboriginal population may achieved through a process of case finding and treatment, using the current best practice guidelines for treatment. This strategy could be employed as an alternative to mass drug administration, and does not rest upon the need for environmental manipulation.

## Supporting information

S1 File Follow up data. (XLSX)

S1 Checklist STROBE checklist. (DOCX)
