# Supplementary material for: Control of chronic Strongyloides stercoralis infection in an endemic community may be possible by pharmacological means alone: Results of a three-year cohort study
Source: PLoS Negl Trop Dis. 2017 Jul 31;11(7):e0005825. doi: 10.1371/journal.pntd.0005825 (PMC5552336; doi:10.1371/journal.pntd.0005825)
Supplement: S1 Checklist — (DOCX) [file pntd.0005825.s002.docx]

| STROBE CHECK LIST  1.  **Title and Abstract**  (*a*) Indicate the study’s design with a commonly used term in the title or the abstract (Abstract Page 1 Line 34) |
| --- |
| (*b*) Provide in the abstract an informative and balanced summary of what was done and what was found (Abstract Page Line 31-46) |

2.

| Introduction | | |
| --- | --- | --- |
| Background/rationale | 2 | Explain the scientific background and rationale for the investigation being reported (Page 2 Line 75-138) |
| Objectives | 3 | State specific objectives, including any pre-specified hypotheses (Page 3 Line 138-148 |

3.

| Methods | | |
| --- | --- | --- |
| Study design | 4 | Present key elements of study design early in the paper (Page 3 Line 138-141) |
| Setting | 5 | Describe the setting, locations, and relevant dates, including periods of recruitment, exposure, follow-up, and data collection (Page 4 Line 155-173) |
| Participants | 6 | (*a*) Give the eligibility criteria, and the sources and methods of selection of participants. Describe methods of follow-up (Page 4 Line 166-173) |
|  |  | (*b*) For matched studies, give matching criteria and number of exposed and unexposed N/A |
| Variables | 7 | Clearly define all outcomes, exposures, predictors, potential confounders, and effect modifiers. Give diagnostic criteria, if applicable (Page 4 177-195) Page 5 Line 222-237, Line 241-253) |
| Data sources/ measurement | 8* | For each variable of interest, give sources of data and details of methods of assessment (measurement).(Page 4 Line 177-183, Page 5Line 246-253) Describe comparability of assessment methods if there is more than one group N/A |
| Bias | 9 | Describe any efforts to address potential sources of bias N/A |
| Study size | 10 | Explain how the study size was arrived at N/A |
| Quantitative variables | 11 | Explain how quantitative variables were handled in the analyses. If applicable, describe which groupings were chosen and why Page 6 Line 256-261. |
| Statistical methods | 12 | (*a*) Describe all statistical methods, including those used to control for confounding (Page 5 Line 255-261) |
|  |  | (*b*) Describe any methods used to examine subgroups and interactions (Page 5 Line 255-261) |
|  |  | (*c*) Explain how missing data were addressed Page 5 Line 259 |
|  |  | (*d*) If applicable, explain how loss to follow-up was addressed N/A |
|  |  | (*e*) Describe any sensitivity analyses N/A |

4.

| Results | | |
| --- | --- | --- |
| Participants | 13* | (a) Report numbers of individuals at each stage of study—eg numbers potentially eligible, examined for eligibility, confirmed eligible, included in the study, completing follow-up, and analysed (Page 7 Line 282-285, Figure 1)) |
|  |  | (b) Give reasons for non-participation at each stage (Figure 1) |
|  |  | (c) Consider use of a flow diagram (Figure 1) |
| Descriptive data | 14* | (a) Give characteristics of study participants (eg demographic, clinical, social) and information on exposures and potential confounders (Page 6 Table 1) |
|  |  | (b) Indicate number of participants with missing data for each variable of interest (Table 3) |
|  |  | (c) Summarise follow-up time (eg, average and total amount) Lines 282-285 |
| Outcome data | 15* | Report numbers of outcome events or summary measures over time Page 7 Line282, 297, Table 2) |
| Main results | 16 | (*a*) Give unadjusted estimates and, if applicable, confounder-adjusted estimates and their precision (eg, 95% confidence interval). Make clear which confounders were adjusted for and why they were included Page 7 Lines 289-293 |
|  |  | (*b*) Report category boundaries when continuous variables were categorized N/A |
|  |  | (*c*) If relevant, consider translating estimates of relative risk into absolute risk for a meaningful time period N/A |
| Other analyses | 17 | Report other analyses done—eg analyses of subgroups and interactions, and sensitivity analyses (Page 8 Line 341-350, Table 4) |

5.

| Discussion | | |
| --- | --- | --- |
| Key results | 18 | Summarise key results with reference to study objectives (Page 9 Lines 354-359) Page 11 Lines 497-503) |
| Limitations | 19 | Discuss limitations of the study, taking into account sources of potential bias or imprecision. Discuss both direction and magnitude of any potential bias Page 10 Line 436-449) |
| Interpretation | 20 | Give a cautious overall interpretation of results considering objectives, limitations, multiplicity of analyses, results from similar studies, and other relevant evidence (Page11 Lines 441-495) |
| Generalisability | 21 | Discuss the generalisability (external validity) of the study results ( Page 9 Lines 363-401) |
| Other information | | |
| Funding | 22 | Give the source of funding and the role of the funders for the present study and, if applicable, for the original study on which the present article is based N/A |
